# Specialist Neurology Involvement and Impact in Immune Checkpoint Inhibitor-Related Neurotoxicity: Experience in a Unified Healthcare System

**DOI:** 10.3390/cancers17243935

**Published:** 2025-12-09

**Authors:** Benjamin Schroeder, Prakrit Prasad, Ope Gbadegesin, Senjuti Gupta, Ricky Frazer, Smilla Heaney, Hester Franks, Cameron Blair, Matthew Stuttard, Clare Barlow, Harriet Cook, Helen Winter, Paolo d’Arienzo, Jake Symington, Yassmeen Radif, Sanketh Rampes, Paul Nathan, Kate Young, Heather Shaw, Aisling Carr, Mark Willis

**Affiliations:** 1 Helen Durham Centre for Neuroinflammatory Diseases, Department of Neurology, University Hospital of Wales, Heath Park, Cardiff CF14 4XN, UK; 2 Department of Psychological Medicine and Clinical Neuroscience, Cardiff University, University Hospital of Wales, Heath Park, Cardiff CF14 4XN, UK; 3 Velindre Cancer Centre, Velindre Road, Cardiff CF14 2TL, UK; 4 Centre for Cancer Sciences, Translational Medical Sciences, Biodiscovery Institute, University of Nottingham, Nottingham NG7 2RD, UK; 5 Department of Oncology, Nottingham University Hospitals NHS Trust, Nottingham City Hospital, Hucknall Road, Nottingham NG5 1PB, UK; 6 Somerset Foundation Trust, Musgrove Park Hospital, Parkfield Drive, Taunton, Somerset TA1 5DA, UK; 7 The Bristol Haematology and Oncology Centre, Horfield Road, Bristol BS2 8ED, UK; 8 Department of Cancer Services, The Royal Marsden Hospital, Fulham Road, London SW3 6JJ, UK; 9 Centre for Neuromuscular Diseases, National Hospital for Neurology and Neurosurgery, Queen Square, London WC1N 3BG, UK; 10 Department of Medical Oncology, Mount Vernon Cancer Centre, Rickmansworth Road, Northwood, Middlesex HA6 2RN, UK; 11 University College London Hospitals, 250 Euston Road, London NW1 2BU, UK; 12 Department of Neuromuscular Disease, Institute of Neurology, University College London, Queen Square, London WC1N 3BG, UK

**Keywords:** immunotherapy, immune-related adverse events, neurotoxicity, neurology

## Abstract

Background: Neurological immune related adverse events (N-irAEs) following immune checkpoint inhibitor (ICI) therapy are associated with significant morbidity and mortality. The early involvement of neurological services is therefore recommended to assist diagnosis and guide management. However, the practical experience of specialist neurology involvement is poorly understood. Methods: A multi-centre, retrospective case note review was performed in a unified healthcare setting in the United Kingdom via predetermined proforma to investigate the involvement and impact of neurology services in this setting. Results: One hundred and nine patients with N-irAE were identified with a median time from ICI treatment to symptom onset of 52 days. Neurology service models, reasons for referral and referral rates varied by centre. Overall, eighty-seven (79.8%) patients (range 52.9–100% by centre) had neurology involvement. Neurology input was associated with younger age (median 67.2 vs. 72.8 years), anatomical location (Central > Peripheral) and severity of neurotoxicity (*p* < 0.001, q < 0.004). Patients with neurology involvement were more likely to undergo specialist investigations: MR imaging (*p* = 0.041, q = 0.043), lumbar puncture (*p* < 0.001, q < 0.004), and neurophysiology (*p* = 0.005, q = 0.007) resulting in a broader range of specific N-irAE diagnoses. Steroids were appropriately prescribed, with second line treatment (Intravenous immunoglobulins/Plasma exchange) associated with neurology involvement. At lower grades (CTCAE ≤ 2), resolution rates were similar in those with or without neurology involvement. At grades 3–4, one-third of patients with neurology involvement had resolution. In a centre with a model of early neurology involvement for all possible N-irAEs the aetiology of the neurological presentation was changed in 63.7%. Conclusions: This study highlights the potential to improve diagnosis and treatment algorithms and therefore patient outcomes through development of uniform N-irAE models of care to support this area of growing clinical need.

## Simple Summary

Immune Checkpoint Inhibitors are revolutionary medications for cancer but can have devastating immune-related side-effects that can injure any part of the nervous system. Within this paper we describe the experience of patients with these side-effects who saw oncologists only, and those who were reviewed by both neurologists and oncologists. Referral to neurology was associated with younger age and more severe side-effects. Those who saw neurologists had more investigations (e.g., lumbar puncture), and received higher doses of steroids as well as more specialised treatments (e.g., intravenous immunoglobulins). A related analysis found that 63.7% of patients with suspected immune-related side-effects were diagnosed with a different neurological condition, when reviewed by a specialist neurologist in a single centre. In conclusion, we suggest the development of uniform models of care across centres to provide equitable and effective management of these conditions.

## 1. Introduction

Immune Checkpoint Inhibitors (ICIs) have revolutionised cancer therapy over the last decade, with significant and durable effects observed across a broad range of malignancies [1]. Despite their clinical efficacy, immune-related adverse events (ir-AE) are commonly observed—predominantly dermatological, endocrine and gastrointestinal toxicities [2]. Neurological immune-related adverse events (N-irAEs) occur less frequently, with severe N-irAEs seen in 1–2% of treated cases [3,4] but are associated with significant morbidity and mortality [5].

N-irAEs can affect any part of the nervous system with a large array of clinical syndromes having been described [6]. The early involvement of a specialist neurology opinion is recommended [7] to help consider the differential diagnoses and direct appropriate investigations and treatment based on analogous *autoimmune* neurological syndromes. In addition, neurologists can aid monitoring of disease recurrence, assess risk of ICI treatment in patients with pre-existing autoimmune disease and participate in multidisciplinary discussions with oncologists, particularly about treatment strategies and re-challenge.

Although some independent centres have established immuno-oncology multi-specialty boards to co-ordinate care [8], the extent of specialist neurology involvement in N-irAE diagnosis and management has not been well described. This study focuses on specialist clinical neurology involvement in N-irAE diagnosis and management in a unified healthcare setting (National Health Service in England and Wales) to explore current access to and impact of neurology opinion in the care of this patient cohort.

## 2. Materials and Methods

Data was collected by retrospective case note review via a consensus proforma from five United Kingdom (UK) neurology/oncology centres: Velindre Cancer Centre/University Hospital of Wales, Cardiff; Bristol Haematology & Oncology Centre, Bristol; Somerset Foundation Trust, Taunton; Nottingham University Hospitals NHS Trust, Nottingham; The Royal Marsden Hospital/National Hospital for Neurology and Neurosurgery (NHNN)/University College London Hospitals (UCLH), London. All neurological immune-related adverse events (N-irAEs) known to each centre occurring between 2019 and October 2024 were included. N-irAEs were defined as new neurological symptoms developing in the context of recent immune-checkpoint inhibitor exposure where non-immune aetiologies had been excluded. In one centre in which the neurology model of care supported early referral of all possible N-irAEs (Royal Marsden Hospital/UCLH/NHNN) a further analysis of final diagnosis in non-N-irAEs was performed. Contributing authors were oncologists and neurologists with experience in the management of immunotherapy toxicity. Ethical approval was obtained from The NHS Health Research Authority (HRA)/Health and Care Research Wales (HCRW), REC: 23/HCRW/0032.

Demographic information, cancer type, staging, and treatment was recorded alongside information on neurological clinical presentation, investigations, management, and clinical outcome (Modified Rankin Scale, mRS) at last follow up. Comparison of corticosteroid doses used an online steroid conversion calculator [9]. “High-dose methylprednisolone” without an explicit dose was taken to be an average of 500–1000 mg, i.e., 750 mg. When a weight was not specified but the dose was weight-based, a weight of 70 kg was assumed. N-irAEs were graded according to the Common Terminology Criteria for Adverse Events (CTCAE) [10]. Resolution was defined as a complete response to treatment back to clinical baseline. Recurrence was defined as worsening of symptoms following complete resolution. Low grade toxicity was defined as CTCAE ≤ 2 and high grade as CTCAE ≥ 3.

Data was analysed in R (v 4.5.0). Statistical tests included: Chi squared test for goodness-of-fit or Fisher’s exact test (if small sample sizes, e.g., individual stratum < 5), Student’s T test for comparison of continuous data between two categorical variables (for parametric data) and Mann–Whitney U test (for non-parametric). Statistical significance was defined as *p* < 0.05. For multiple comparisons, False Discovery Rate correction was applied.

## 3. Results

### 3.1. Cohort Characteristics

**Figure 1:**
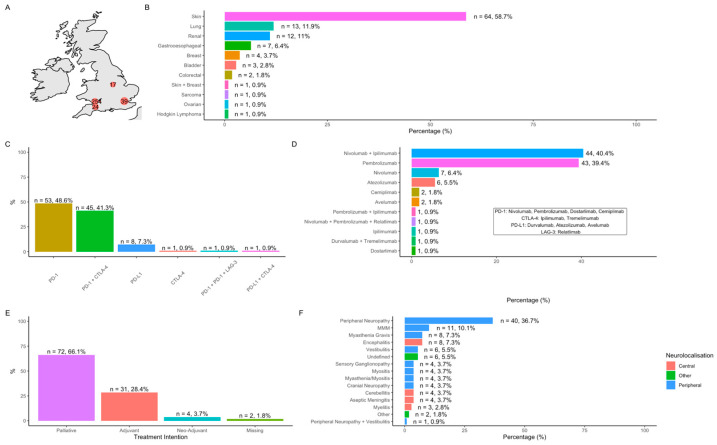
Cohort characteristics. (**A**) Number of cases per participating centre; (**B**) site of primary cancer; (**C**) ICI classes and combinations; (**D**) individual ICI and combinations; (**E**) treatment intent; (**F**) frequency of observed NirAEs.

One hundred and nine patients with neurological immune-related adverse events (N-irAEs) were identified: London, *n* = 39 (35.8%); Cardiff, *n* = 25 (22.9%), Taunton, *n* = 24 (22%); Nottingham, *n* = 17 (15.6%); Bristol, *n* = 4 (3.7%) (Figure 1A). Sixty-four (58.7%) patients were male with a median age of 68.7 years (range 16.9–93). Overall cohort characteristics are demonstrated in Supplementary Table S1. The most common site of primary malignancy was skin (*n* = 64, 58.7%) with an additional patient having skin and breast cancer (Figure 1B) and metastatic disease observed in 78.0% (*n* = 85). By ICI class, anti-PD-1 agents (*n* = 99, 90.8%) were the most commonly administered, either alone or in combination (Figure 1C,D). The majority of patients (66.1%, *n* = 72) received ICI with palliative intent (Figure 1E).

### 3.2. Neurological Immune-Related Adverse Events (N-irAEs)

#### 3.2.1. Clinical Phenotypes

Frequency of observed N-irAEs is demonstrated in Figure 1F and patient characteristics summarised in Table 1. Overall, peripheral nervous system N-irAE (*n* = 83, 80.6%) outnumbered central N-irAEs (*n* = 20, 19.4%). Peripheral neuropathy/neuritis (*n* = 40, 36.7%) was the most commonly observed individual N-irAE followed by encephalitis/cerebellitis (*n* = 12, 11%) and the overlap syndrome of myasthenia gravis/myositis/myocarditis (‘MMM syndrome’, *n* = 11, 10.1%) (Figure 1F). CTCAE grades of neurotoxicity included: grade 1: *n* = 5, (4.6%); grade 2: *n* = 42, (38.5%) grade 3: *n* = 38, (34.9%), grade 4: *n* = 18, (16.5%); grade 5: *n* = 6 (5.5%).

#### 3.2.2. Time to N-irAE

**Figure 2:**
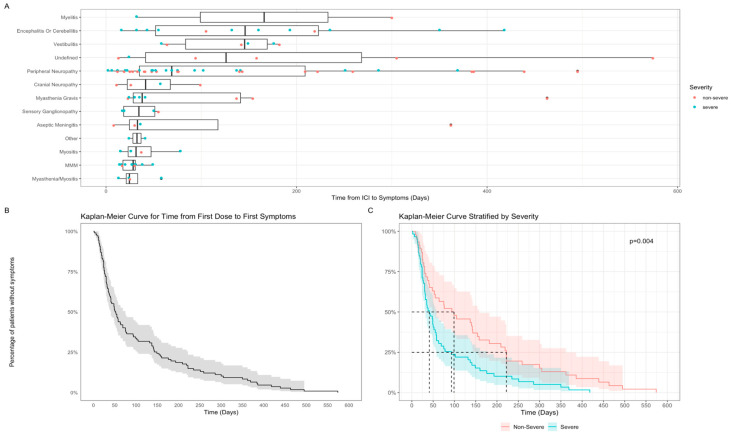
Time from first ICI exposure to NirAE. (**A**) Box and whisker plot—time from ICI treatment to symptom onset grouped by specific N-irAE presentations; (**B**) Kaplan–Meier survival curve for time to N-irAE symptom onset from ICI treatment (whole cohort); (**C**) Kaplan–Meier survival curve for time to N-irAE symptom onset by CTCAE severity grade.

Time from first ICI exposure to symptom onset varied. Overall, median time to N-irAE from first dose was 52 days (range: 2–574). The fastest onset was myasthenia gravis and myositis with a median time of 24.5 days (range: 13–58) and the longest being myelitis with a median time of 166 days (range: 32–300) (Figure 2A). Time to first symptoms differed by N-irAE severity; high grade (CTCAE grade ≥ 3) presentations occurred with median onset of 41 days (*n* = 61, range 2–418) compared to low grade (CTCAE ≤ 2) (*n* = 47, median 94 days, 8–574) (*p* = 0.004, q = 0.007) (Figure 2B,C).

### 3.3. Neurology Service Models and Reasons for Referral

**Figure 3:**
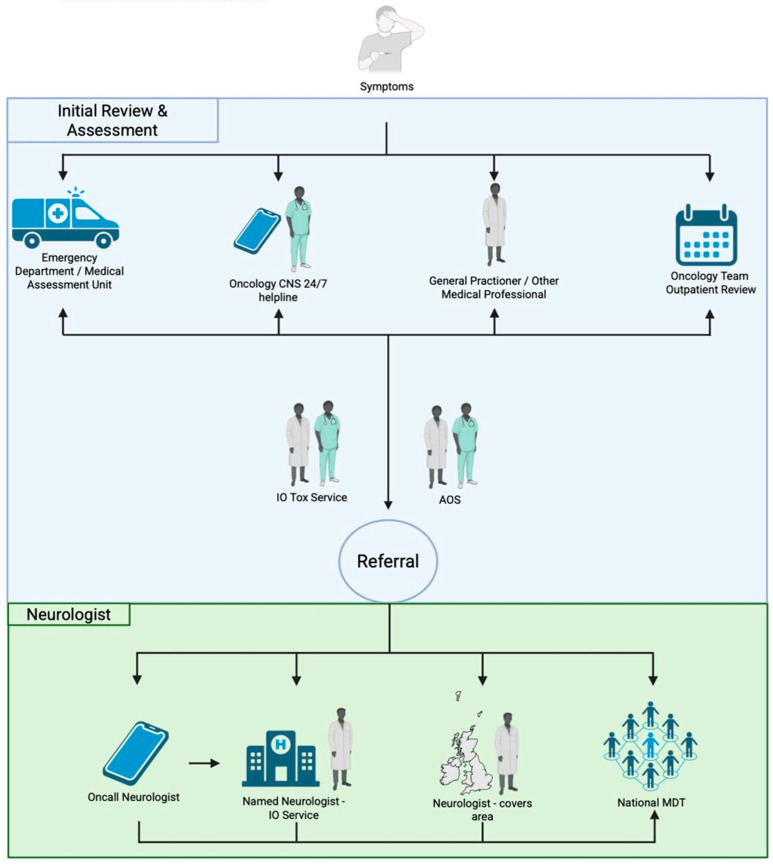
Access to Oncology/Neurology services. Initial review and assessment—Patients may present to a number of locations at symptom onset (i) emergency department/acute medical unit; (ii) via contact with oncology specialist nurse/24 h helpline; (iii) via general practitioner; (iv) via routine outpatient oncology team review. Patients may also flow between these different locations. Following initial presentation, further input may occur via the acute oncology service (AOS) and/or the local immune-oncology (IO) toxicity service.

Access to Acute Oncology Services was similar across all sites (Figure 3). Following the recognition of N-irAE, reasons for onward neurology referral differed by site as did local service provision. In office hours (9 a.m.–5 p.m.), patients were referred to a named neurologist as part of an established immunotherapy toxicity service (two centres), or to the local, or on call general neurology service (three centres). On call neurology services were utilised by all centres for out of hours or time-critical presentations.

No specific neurology referral guidelines existed for any of the centres in this study. Reasons for referral to neurology varied by centre but included: (i) presentations with the potential for rapid deterioration requiring transfer to an acute hospital with specialist neurology care and intensive care facilities, e.g., MMM, Guillain-Barré syndrome, encephalitis; (ii) for specialist treatment (Intravenous immunoglobulin, IVIg; Plasma exchange, PLEX); (iii) for detailed clinical phenotyping to aid neuroanatomical localisation and formulation of differential diagnoses; (iv) discussion about relevant next investigations; (v) high grade (CTCAE ≥ 3) neurological presentations (vi) patients not responding to first line treatment, relapsing on lower doses of steroids, or recurrence of symptoms.

### 3.4. Specialist Neurology Involvement

**Figure 4:**
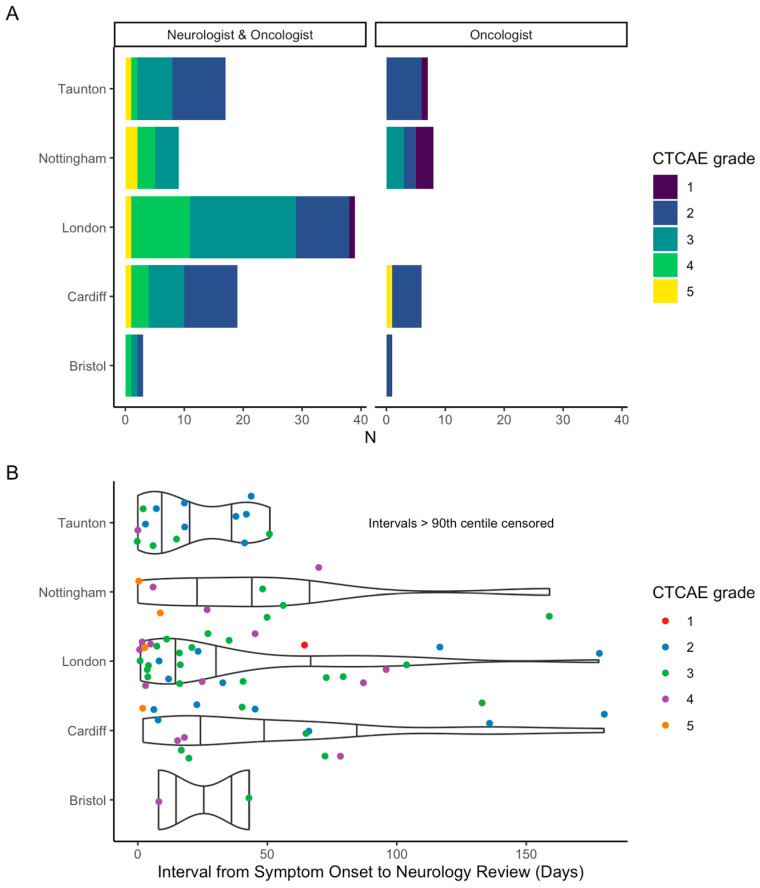
(**A**) Neurology involvement by participating site, stratified by CTCAE grade; (**B**) violin and scatter plot of interval between symptom-onset and neurology review per centre. N.B. given large range of interval, >90th centile omitted from figure.

Eighty-seven (79.8%) patients had a neurologist’s involvement. Neurologist involvement varied from in-person review (*n* = 76, 87.4%), direct correspondence via email or telephone (*n* = 9, 10.3%), or multi-speciality team discussion (*n* = 2, 2.3%). Rate of neurologist involvement varied between centres: London (*n* = 39, 100%), Cardiff (*n* = 19, 76%), Taunton (*n* = 17, 70.8%), Nottingham (*n* = 9, 52.9%), and Bristol (*n* = 3, 75%) (*p* < 0.001) (Figure 4A).

Time to neurologist’s review from symptom onset also varied by site: Taunton (*n* = 16), median 18 days (range 0–783); London (*n* = 36) median 22 days (range 1–460); Bristol (*n* = 2) median 26 days (range 8–43); Cardiff (*n* = 18) median 43 days (range 2–854); and Nottingham (*n* = 9) median 48 days (range 0–159) (Figure 4B). Data was not available for time to neurologist’s review from date of referral.

#### 3.4.1. Factors Associated with Neurology Involvement

Patients receiving neurologist’s input tended to be younger (neurologist’s involvement: median 67.2 years (IQR: 19.5) vs. no neurologist’s involvement: 72.8 years (IQR: 21)) and involvement differed by anatomical localisation. Of the centrally localising N-irAEs, all 20 patients (100%) were referred to neurology. Of those with neuromuscular junction disease, 91.3% (*n* = 21) had neurologist’s involvement, whilst only 73.3% (*n* = 44) of peripheral N-irAEs were referred.

Maximal (CTCAE) grade of N-irAE was also associated with neurologist’s involvement. Of those patients with grade 1 toxicity, 20% (1/5) were referred to neurology; grade 2, 66.7% (28/42), grade 3, 92.1% (35/38), grade 4, 100% (18/18), and grade 5, 83.3% (5/6)) (*p* < 0.001, q < 0.004). Of patients with severe N-irAE (CTCAE ≥ 3) 93.5% (58/62) had a neurologist’s opinion.

#### 3.4.2. Effect of Neurologist’s Involvement on Investigations and Diagnosis

The frequency of requested investigations differed depending on whether a neurologist was involved in patient care: lumbar puncture (LP)—neurologist, 35 (40.2%) vs. no-neurologist 0 (0%) (*p* < 0.001, q < 0.004); MRI—71 (82.6%)) vs. 13 (59%) (*p* = 0.041, q = 0.043); neurophysiology (nerve conduction studies/electromyography)–45 (51.7%) vs. 4 (18.2%) (*p* = 0.005, q = 0.007).

Directed tissue biopsy was uncommonly performed (*n* = 5, 4.8%) with all cases occurring in one centre (NHNN/UCLH) at an early stage of neurologist involvement in N-irAE to improve understanding of disease pathogenesis: sural nerve biopsy (neuropathy/neuritis, *n* = 3); muscle biopsy (myasthenia gravis and myositis *n* = 1, MMM syndrome, *n* = 1).

Following these investigations, the range of eventual diagnoses was more extensive in those patients reviewed by neurology with fourteen different individual N-irAE diagnoses identified in those where a neurologist was involved vs. five in those where oncology alone had been involved in patient care.

#### 3.4.3. Effect of Neurologist’s Involvement on Clinical Management

Ninety-eight (89.9%) patients received steroids; seventeen (15.6%) received IVIg, thirteen (11.9%) underwent PLEX, and ten (9.2%) did not receive any treatment. Of those who received steroids, (94/98, 96%, Table 2) had initial prednisolone equivalent doses available.

**Figure 5:**
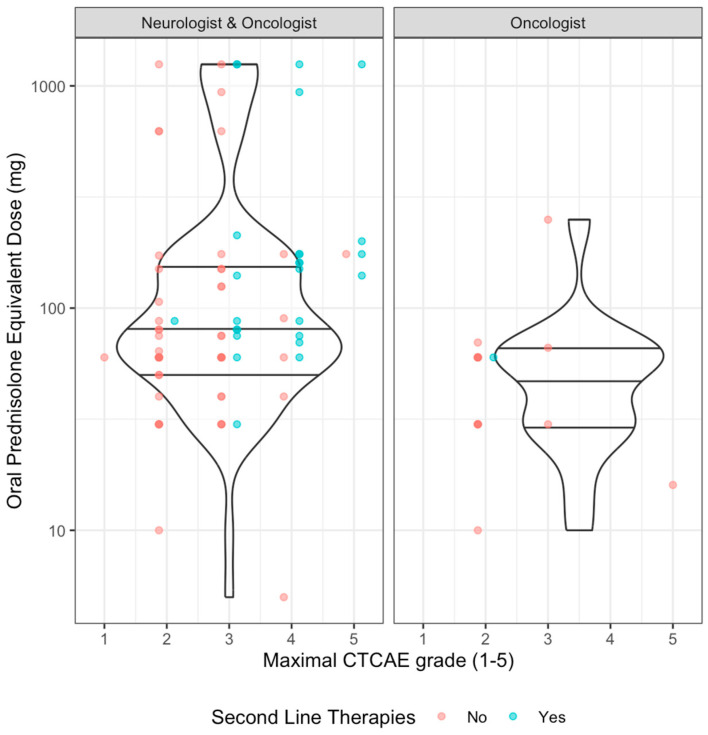
Induction steroid dose by CTCAE grade and neurological involvement. Scatter plot with violin plot (quartiles as horizontal lines). Colours by whether second line immunomodulating/suppressive agents were used.

Steroid use was associated with CTCAE grade, with overall steroid use more frequent in higher grades of N-irAE: grade = 1, *n* = 1/5 (20%); grade = 2: *n* = 39/42 (92.9%); grade = 3, *n* = 34/38 (89.5%); grade 4, *n* = 18/18 (100%); grade 5: *n* = 6/6 (100%). Steroid prescribing was associated with neurologist’s involvement—81/87 (93%) patients who had a neurologist’s opinion received steroids, compared to 17/22 (77.2%) patients who received steroids without neurology input (*p* = 0.043, q = 0.043). When comparing by severity of toxicity, in those with lower grade (CTCAE ≤ 2) initial median steroid dose was comparable in those with or without neurologist’s involvement but with a wider range of doses used in those with neurology input (Table 2, Figure 5). At higher grades (≥CTCAE grade 3) comparison between groups was limited due to small numbers in the oncology-only group but overall patients received higher doses commensurate with established guidelines (Figure 5).

IVIg was uniquely administered with neurologist’s input (*n* = 17, 100%). PLEX was used in 13 cases, 12 of which had a neurologist’s opinion (92.3%) (Table 3). Indications for IVIg included: myasthenia/myositis, *n* = 2 (50% of those with this diagnosis); myasthenia gravis, *n* = 4 (50%); sensory ganglionopathy, *n* = 2 (50%); MMM, *n* = 3 (27.3%); peripheral neuropathy/neuritis, *n* = 4, (9.8%); encephalitis or cerebellitis, *n* = 1, (8.3%). PLEX was administered in the following N-irAEs: encephalitis/cerebellitis, *n* = 1 (8.3%); MMM, *n* = 4 (36.3%); myasthenia gravis, *n* = 3 (37.5%); peripheral neuropathy, *n* = 1 (2.5%); myelitis, *n* = 1 (33.3%); myasthenia/myositis, *n* = 1 (25%); myositis, *n* = 1 (25%), and other, *n* = 1 (50%).

Of all the patients (*n* = 14) that were prescribed a second line immunosuppressant, 13 (92.9%) had high grade toxicity, all of which had neurologist’s input (Table 3). With steroid dose, where data was available, concurrent bone protection was prescribed in 54.4% cases when a neurologist was involved compared with 16.7% where a neurologist was not. Similarly, gastric protection and *Pneumocystis jirovecii pneumonia* (PJP) prophylaxis was more common in patients where a neurologist was involved; 92% vs. 71.4%, and 23.9% vs. 8.3%, respectively (with available data).

#### 3.4.4. Effect of Neurologist’s Involvement on Clinical Outcomes

Data on N-irAE symptom resolution was available for 85/109 (78%) patients (Table 4). Overall, 25/70 (36%) patients had resolution with neurologist’s input compared to 7/15 (47%) without. Comparison was limited due to a lack of available data but resolution rates at lower grades appeared similar in both groups. Approximately one-third of patients with grade 3 or 4 toxicity experienced resolution after neurologist’s involvement (Table 4).

Recurrence data was available for 94/109 (86%) patients, although the dataset was too small to allow comparison between groups (Table 4). Overall, 11 (11.7%) patients experienced recurrence. Rechallenge data was available for 106/109 (97.2%) patients with 20/106 (18.8%) rechallenged with ICI, but only in 7 patients who experienced higher grade toxicity (Table 4).

#### 3.4.5. Analysis of Immuno Oncology-Neurotoxicity Service

In 2/5 centres there was an established Immuno Oncology-neurotoxicity service involving early referral to a specific consultant neurologist. In one centre (UCLH-NHNN) this was set up in 2019 and site-specific data was available on all referrals to that service from oncologists from surrounding hospitals over a 4-year period. The NHNN immunotherapy neurotoxicity service accepted all patients in whom N-irAE was under consideration for prompt specialist neurological clinical opinion and directed investigation when required.

Between 2019 and 2023 the NHNN IO-Neurotoxicity service saw 102 patients referred by oncologists from 4 major oncology centres (Supplementary Table S2). A total of 36.3% of the patients were diagnosed with N-irAEs reflecting the spectrum described above and included in previous analysis. However, a large proportion were ultimately diagnosed with non-immune related neurology (*n* = 65, 63.7%).

Of those patients who were diagnosed with non-immune-related neurological diseases the most common group was that of alternative neurological diagnoses (*n* = 27), followed by non-ICI iatrogenic (*n* = 17), infiltrative (*n* = 6), non-neurological irAE (*n* = 6), paraneoplastic (*n* = 2) and other (*n* = 6). The alternative neurological diagnoses included: non-inflammatory neuropathy or radiculopathy including pre-existing idiopathic axonal neuropathy, compressive mononeuropathies (related to cancer associated weight loss) and diabetic neuropathy (*n* = 12), migraine (*n* = 6), dementia/mild cognitive impairment (*n* = 2), prion disease (*n* = 1), idiopathic Parkinson’s disease (*n* = 1), essential tremor (*n* = 1), Bell’s palsy (*n* = 1), Benign Paroxysmal Positional Vertigo (*n* = 1), RyR1 myopathy (*n* = 1), and isolated, microvascular third nerve palsy presenting with diplopia and unilateral non-fatigable ptosis (*n* = 1). Iatrogenic cases included symptoms related to platinum or taxane chemotherapy (CIPN) (*n* = 5), steroid toxicity including psychosis and steroid-myopathy (*n* = 3), radiotherapy-associated facial nerve palsy (*n* = 2), post-radiotherapy plexopathy (*n* = 1), and six others.

A change from presumed N-irAE to an alternative neurological diagnosis/alternative diagnosis with confirmation on supportive investigation resulted in treatment change for all patients in whom ICI therapy had been paused (100%) and corticosteroids commenced prior to referral.

## 4. Discussion

This study across five UK cancer centres, identified differences in neurology service models including variation in the reasons and rates of referral. Commonalities across all centres included higher rates of neurologists’ involvement in younger patients with higher CTCAE grade toxicities and central nervous system manifestations. Impact of neurologists’ involvement included utilisation of more directed diagnostic investigations, higher corticosteroid dosing and greater use of second- and third-line immunosuppression. One third of patients with CTCAE ≥ 3 N-irAE achieved complete resolution of neurological deficit.

Given the exponential rise in use of ICI and the subsequent increasing incidence of N-irAE, there is growing importance for the role of neurologists in this setting. Whilst neurologists’ involvement is recommended in international guidelines and their input is recognised as essential by Oncology colleagues [11], there is no consensus on neurology referral and limited practical understanding of their current involvement.

Two centres had access to a neurologist as part of an immuno-oncology multidisciplinary service, with others largely dependent on general neurology on-call services. These different service models were reflected in the time from symptom onset to neurologist’s review, with wide variation seen across centres. Of note, although the main indication for immunotherapy in this study was ‘palliative’, it should be noted that such patients in a melanoma setting with combination ICI therapy have significant long term survival rates and therefore the morbidity and mortality associated with N-irAEs is of increasing concern [12].

The range and characteristics of N-irAEs described in this study are similar to those previously reported in large single centre and national cohort studies [6,13]. With neurologist’s involvement, patients were more likely to have specialist investigations performed (MR imaging, lumbar puncture, neurophysiology) demonstrating the additional diagnostic methodology employed by neurologists to ensure an accurate diagnosis—the wider range of specific N-irAE diagnoses in those with neurologists’ involvement reflects this. The importance of thorough diagnostic evaluation was further demonstrated in a sub-cohort of patients referred for assessment of potential N-irAE in one centre where the original diagnosis of N-irAE was overturned in 63.7% of referrals, consistent with previous findings [8]. Ensuring an accurate diagnosis in this context was essential to ensure appropriate management and to avoid potential iatrogenic harm from immunosuppressive therapy and possible associated adverse cancer outcomes.

Corticosteroid dosing as first-line management for irAEs in this study reflected standard practice. Neurologists’ involvement was associated with the use of second line treatment with IVIg and PLEX. This is explained by the association between neurologists’ involvement and more severe grades of toxicity as well as neurologists’ experience in the use of these treatments with analogous *autoimmune* presentations, and as recommended by established guidelines [14]. Interestingly, neurologists were also more likely to prescribe concurrent medications with steroid use (bone and gastric protection, and PJP prophylaxis) demonstrating neurologists’ experience in the use of administering immunosuppressive treatment in a long-term setting reflecting the gradual rate of recovery observed in neurological disorders.

The neurologists in our study group have an interest in N-irAE so observations reported here may not reflect national level provision of neurology support. Smaller centres may have less access to a neurology opinion than those included in this study. Greatest oncology expertise in management of irAEs comes from experience in melanoma treatment which is reflected in skin cancer predominance in our cohort. Nevertheless, differences in service delivery were observed. Acute oncologists have gained much skill and experience in prompt recognition and management of a broad spectrum of organ-specific immunotherapy complications and considerable expertise in their management, and immunotherapy-related toxicity services have been established in major centres [8,15,16]. This study suggests that there may be further benefit to patients and outcomes from a streamlined model for neurology referral, accessible to all oncologists using immunotherapies. Standardised guidelines for early referral to neurology may facilitate more accurate diagnoses and direct either escalation of immunosuppression or alternative management options; alongside established general N-irAE guidelines (e.g., ESMO) the development of toxicity-specific (e.g., MMM) guidelines will also be important. How these services are delivered and directed to appropriately skilled sub-specialist neurologists in an equitable fashion across all cancer treatment centres will require a coordinated network approach, with further benefit from national specialist advisory group oversight (national multidisciplinary team meeting) for more complex or treatment refractory cases. The development of neurology sub-specialisation in this area will be of importance for future service development, with not all general neurologists having the necessary expertise to support patients with N-irAE. This will be especially important as management strategies evolve with better understanding of underlying disease pathogenesis, which may be found to differ from their analogous *autoimmune* counterparts.

We acknowledge that there are several confounding factors to consider including the fact that neurology service models and patterns of referral may have varied during the time course of this retrospective study. Given the rapid evolution of the field of ICI-neurotoxicity we were unable to provide direct comparisons on the influence of neurology input on patient outcome. However, these data suggest that oncologists utilise neurologists in a proportion of cases with impact on diagnosis and treatment, and when neurology input is required, it should be prompt and responsive.

## 5. Conclusions

Here we show how access to and utilisation of neurology specialist input into the care of N-irAEs differs by geographical area within a single, unified healthcare system in a high-income country.

In summary:

- Models of neurology services, reasons and rates of referral differ across the UK;
- Neurology opinion was associated with younger age and severity of neurotoxicity;
- Neurology opinion was universally sought for CNS toxicity (more than PNS) even though PNS complications were more common;
- Neurologists were more specific in their utilisation of investigations with a more diverse range of N-irAEs diagnoses with the added potential for identifying alternative diagnoses;
- Specialist second-line treatments were associated with neurology involvement.

These data could inform future service development, including referral, investigation and management pathways to facilitate geographically equitable access to optimal clinical care of N-irAEs.

## Figures and Tables

**Table 1:** Patient Characteristics by N-irAE: Data summarising the average age, and frequencies of various factors stratified by individual neurological immune related adverse events (neurotoxicity/N-irAE). ^1^ median (interquartile range) * number of patients with outcome, where data is available.

Neurotoxicity	N	Age ^1^	Sex (M)	ICI	Cancer	MRI	CSF	NeuroPhy	Management	Outcome
Peripheral Neuropathy/Neuritis	40	67 (58, 77)	25	PD-1: 39 PD-L1: 0 CTLA-4: 20 LAG-3: 1	Skin: 28 GI: 5 Renal: 3 Lung: 3 Breast: 1 Other: 1	Yes: 26 No: 13	Yes: 13 No: 27	Yes: 29 No: 11	Steroids: 32 IVIg: 4 PLEX: 1	Resolution: 12 * Recurrence: 3 * Rechallenge: 12 *
MMM	11	77 (61, 81)	9	PD-1: 10 PD-L1: 1 CTLA-4: 3 LAG-3: 0	Skin: 8 Renal: 1 GI: 1 Bladder: 1	Yes: 7 No: 4	Yes: 0 No: 11	Yes: 1 No: 10	Steroids: 11 IVIg: 3	Resolution: 2 * Recurrence: 0 Rechallenge: 1 *
Encephalitis or Cerebellitis	12	64 (57, 73)	5	PD-1: 7 PD-L1: 1 CTLA-4: 2 LAG-3: 0	Skin: 5 Lung: 4 Renal: 2 Other: 1	Yes: 12	Yes: 10 No: 2	Yes:1 No: 11	Steroids: 11 IVIg: 1 PLEX: 4	Resolution: 5 * Recurrence: 2 Rechallenge: 1
Myasthenia Gravis (isolated)	8	78 (63, 86)	5	PD-1: 6 PD-L1: 1 CTLA-4: 1 LAG-3: 0	Skin: 5 Renal: 1 Bladder: 1 CRC: 1	Yes: 6 No: 2	Yes: 0 No: 8	Yes: 4 No: 4	Steroid: 7 IVIg: 4 PLEX: 3	Resolution: 4 Recurrence: 1 * Rechallenge: 0
Vestibulitis	6	63 (54, 67)	3	PD-1: 7 PD-L1: 1 CTLA-4: 4 LAG-3: 0	Skin: 3 Renal: 2 Lung: 1	Yes: 6	Yes: 1 No: 5	Yes: 1 No: 5	Steroid: 5 IVIg: 0 PLEX: 0	Resolution: 1 Recurrence: 0 * Rechallenge: 0
Unclear	6	73 (61, 83)	3	PD-1: 4 PD-L1: 2 CTLA-4: 0 LAG-3: 0	Skin: 2 Lung: 2 Renal: 1 Breast: 1	Yes: 5 No: 1	Yes: 0 No: 6	Yes: 2 No: 4	Steroid: 6 IVIg: 0 PLEX: 0	Resolution: * Recurrence: 2 Rechallenge: 2
Sensory Ganglionopathy	4	75 (66, 79)	3	PD-1: 4 PD-L1: 0 CTLA-4: 3 LAG-3: 0	Skin: 4	Yes: 4	Yes: 2 No: 2	Yes: 4	Steroid: 4 IVIg: 2 PLEX: 0	Resolution: 1 Recurrence: 0 Rechallenge: 0
Aseptic Meningitis	4	50 (33, 60)	1	PD-1: 4 PD-L1: 0 CTLA-4: 3 LAG-3: 0	Skin: 3 GI: 1	Yes: 4	Yes: 3 No:1	Yes: 0 No: 4	Steroid: 4 IVIg: 0 PLEX: 0	Resolution: 3 Recurrence: 1 Rechallenge: 1
Cranial Neuropathy	4	65 (50, 72)	1	PD-1: 3 PD-L1: 1 CTLA-4: 1 LAG-3: 0	Skin: 1 Breast: 2 Bladder: 1	Yes: 3 No: 1	Yes: 1 No: 3	Yes: 0 No: 4	Steroid: 4 IVIg: 0 PLEX: 0	Resolution: 0 * Recurrence: 0 Rechallenge: 1
Myasthenia Gravis and Myositis	4	73 (70, 76)	4	PD-1: 4 PD-L1: 0 CTLA-4: 3 LAG-3: 0	Skin: 2 Lung: 2	Yes: 2 No: 2	Yes: 0 No: 4	Yes: 3 No: 1	Steroid: 4 IVIg: 2 PLEX: 1	Resolution: 1 * Recurrence: 0 Rechallenge: 1
Myositis	4	85 (74, 88)	2	PD-1: 3 PD-L1: 1 CTLA-4: 1 LAG-3: 0	Skin: 2 Renal: 2	Yes: 3 No: 1	Yes: 0 No: 4	Yes: 2 No: 2	Steroid: 4 IVIg: 0 PLEX: 1	Resolution: 2 * Recurrence: 0 Rechallenge: 4
Myelitis	3	76 (55, 77)	1	PD-1: 3 PD-L1: 0 CTLA-4: 0 LAG-3: 0	Skin: 1 Lung: 1 CRC: 1	Yes: 3 No: 0	Yes: 3 No: 0	Yes: 0 No: 3	Steroid: 3 IVIg: 0 PLEX: 1	Resolution: 0 Recurrence: 1 * Rechallenge: 0
Other	2	58 (55, 61)	1	PD-1: 1 PD-L1: 1 CTLA-4: 1 LAG-3: 0	Breast: 1 Other: 1	Yes: 2 No: 0	Yes: 2 No: 0	Yes: 1 No: 1	Steroid: 2 IVIg: 1 PLEX: 1	Resolution: 1 Recurrence: 1 * Rechallenge: 0
Peripheral neuropathy and vestibulitis	1	68.4	1	PD-1: 1	Skin: 1	Yes: 1	No: 1	Yes: 1	Steroid: 1 IVIg: 0 PLEX: 0	Resolution: * Recurrence: 0 Rechallenge: 0

**Table 2:** N-irAE management (steroids) by grade of toxicity and Neurologist’s involvement. Data presented as: count/count who received the treatment at that CTCAE grade. Intravenous Immunoglobulins (IVIg), Plasma Exchange (PLEX), Steroid Sparing Agents (SSA).

	Steroids		Steroid Dose (Median, Range)	
	Neurologist and Oncologist (*n* = 87)	Oncologist only (*n* = 22)	Neurologist and Oncologist (*n* = 87)	Oncologist only (*n* = 22)
	Yes (*n* = 81)	Yes (*n* = 17)		
Grade 1	1/1 (100%)	0/4 (0%)	1/1, 60 (60–60)	0/0
Grade 2	26/28 (92.9%)	13/14 (92.9%)	26/26, 60 (10–1240)	12/13 60 (10–70)
Grade 3	31/35 (88.6%)	3/3 (100%)	30/31, 77.5 (30–1250)	3/3 66.3 (30–250)
Grade 4	18/18 (100%)	0/0	16/18, 120 (5–1250)	0/0
Grade 5	5/5 (100%)	1/1 (100%)	5/5, 175 (140–1250)	1/1, 16 (16–16)

**Table 3:** N-irAE management (IVIg/PLEX/SSA/Biologic) by grade of toxicity and Neurologist’s involvement. Steroid dose = valid dose (n)/total n with steroids at that grade, med (range).

	IVIg		PLEX		SSA/Biologic	
	Neurologist and Oncologist (*n* = 87)	Oncologist only (*n* = 22)	Neurologist and Oncologist (*n* = 87)	Oncologist only (*n* = 22)	Neurologist and Oncologist (*n* = 87)	Oncologist only (*n* = 22)
Treatment received	N = 17	N = 0	N = 12	N = 1	N = 13	N = 1
Grade 1	0/1	0/4	0/1	0/4	0/1	0/4
Grade 2	1/28 (3.6%)	0/14	0/28	1/14 (7.1%)	0/28	1/14 (7.1%)
Grade 3	5/35 (14.3%)	0/3	2/35 (5.7%)	0/3	8/35 (22.9%)	0/3
Grade 4	9/18 (50%)	0/0	7/18 (38.9%)	0/0	4/18 (22.2%)	0/0
Grade 5	2/5 (40%)	0/1	3/5 (60%)	0/1	1/5 (20%)	0/1

**Table 4:** Resolution, recurrence, and rechallenge rates by grade of toxicity and neurologist’s involvement. Data presented as count fitting that criterion/count by that CTCAE grade (percentage of those with valid data for entire neurology or oncology only cohorts; percentage within that stratum of condition and CTCAE grade).

	Resolution		Recurrence		Rechallenge	
	Neurologist and Oncologist (*n* = 87)	Oncologist only (*n* = 22)	Neurologist and Oncologist (*n* = 87)	Oncologist only (*n* = 22)	Neurologist and Oncologist (*n* = 87)	Oncologist only (*n* = 22)
Data available	N = 70 (80.5%)	N = 15 (68.2%)	N = 77 (88.5%)	N = 17 (77.3%)	N = 85 (97.7%)	N = 21 (95.4%)
Grade 1	1/1 (1.4%, 100%)	2/3 (13.3%, 50%)	0/1	0/3	0/1	2/4 (9.5%, 50%)
Grade 2	10/19 (14.3%, 52.6%)	4/8 (26.7%, 50%)	2/27 (2.6%, 7.4%)	2/12 (11.8%, 16.7%)	3/27 (3.5%, 11.1%)	8/13 (38.1%, 61.5%)
Grade 3	8/29 (11.4%, 27.6%)	1/3 (6.7%, 33.3%)	5/29 (6.5%, 17.2%)	0/1	5/35 (5.9%, 14.3%)	1/3 (4.8%, 33.3%)
Grade 4	6/17 (8.6%, 35.3%)	0/0	2/17 (2.6%, 11.8%)	0/0	1/17 (1.2%, 5.9%)	0/0
Grade 5	0/4 (0%, 0%)	0/1	0/3	0/1	0/5	0/1

## Data Availability

The raw data supporting the conclusions of this article will be made available by the authors on request.

## Supplementary Materials

The following supporting information can be downloaded at: https://www.mdpi.com/article/10.3390/cancers17243935/s1, Table S1. Overall cohort characteristics. Table S2. Referrals for possible Neurological Immune Related Adverse Events to a Tertiary Neurology Service.
