# Supplementary material for: Specialist Neurology Involvement and Impact in Immune Checkpoint Inhibitor-Related Neurotoxicity: Experience in a Unified Healthcare System
Source: Cancers (Basel). 2025 Dec 9;17(24):3935. doi: 10.3390/cancers17243935 (PMC12730614; doi:10.3390/cancers17243935)
Supplement: Supplementary file 1 [file cancers-17-03935-s001.zip › cancers-3958253-supplementary.pdf]

Table S1. Overall cohort characteristics.

Pt	Age	Sex	Cancer	Metastatic	ICI	ICI class	Other irAE	Neurology involved	Neurotoxicity	Max grade
1	53.9	Female	renal	Yes	nivolumab + ipilimumab	PD-1 + CTLA-4	Scleritis, Pneumonitis, Adrenal Insuff.	Yes	vestibulitis	2
2	64.1	Male	bladder	Yes	atezolizumab	PD-L1		No	cranial neuropathy	2
3	58.4	Male	gastrooesophageal	Yes	pembrolizumab	PD-1		No	peripheral neuropathy	2
4	78.5	Female	lung	Yes	pembrolizumab	PD-1		Yes	encephalitis or cerebellitis	3
5	76.1	Female	lung	No	pembrolizumab	PD-1		Yes	peripheral neuropathy	2
6	83.6	Male	skin	Yes	nivolumab + ipilimumab	PD-1 + CTLA-4		No	peripheral neuropathy	2
7	78.3	Male	gastrooesophageal	Yes	pembrolizumab	PD-1		Yes	peripheral neuropathy	2
8	86.4	Female	skin	No	pembrolizumab	PD-1		No	mmm	5
9	64.9	Female	renal	Yes	nivolumab + ipilimumab	PD-1 + CTLA-4		Yes	encephalitis or cerebellitis	3
10	76.6	Female	lung	Yes	pembrolizumab	PD-1	Cholecystitis	Yes	myelitis	2
11	91.2	Male	bladder	Yes	atezolizumab	PD-L1		Yes	myasthenia gravis	2
12	16.9	Male	gastrooesophageal	Yes	pembrolizumab	PD-1		Yes	aseptic meningitis	2
13	53.4	Female	renal	Yes	nivolumab + ipilimumab	PD-1 + CTLA-4		Yes	peripheral neuropathy	2
14	75.4	Male	skin	Yes	pembrolizumab	PD-1		Yes	peripheral neuropathy	2
15	58.5	Female	lung	Yes	pembrolizumab	PD-1		Yes	encephalitis or cerebellitis	3
16	68.7	Male	renal	Yes	nivolumab + ipilimumab	PD-1 + CTLA-4		Yes	encephalitis or cerebellitis	3
17	83.7	Male	bladder	Yes	avelumab	PD-L1		Yes	mmm	5
18	79.6	Male	skin	No	pembrolizumab	PD-1		Yes	mmm	4
19	75.3	Male	gastrooesophageal	Yes	pembrolizumab	PD-1		Yes	peripheral neuropathy	3
20	53.9	Female	renal	Yes	nivolumab + ipilimumab	PD-1 + CTLA-4	Pneumonitis, Scleritis	Yes	vestibulitis	4
21	61.3	Male	lung	No	atezolizumab	PD-L1		Yes	vestibulitis	4
22	83.8	Female	renal	Yes	pembrolizumab	PD-1		Yes	myositis	2
23	72.6	Female	lung	Yes	atezolizumab	PD-L1		Yes	encephalitis or cerebellitis	3
24	84.8	Male	gastrooesophageal	No	nivolumab	PD-1		No	peripheral neuropathy	2
25	58.4	Male	gastrooesophageal	Yes	pembrolizumab	PD-1		No	peripheral neuropathy	2
26	52.0	Female	skin	Yes	nivolumab + ipilimumab	PD-1 + CTLA-4		Yes	aseptic meningitis	2
27	68.9	Female	skin	Yes	nivolumab + ipilimumab	PD-1 + CTLA-4		Yes	aseptic meningitis	2
28	52.1	Female	skin	Yes	nivolumab + ipilimumab	PD-1 + CTLA-4		Yes	peripheral neuropathy	3
29	73.1	Male	skin	Yes	nivolumab	PD-1		Yes	encephalitis or cerebellitis	3
30	39.5	Male	skin	Yes	nivolumab + ipilimumab	PD-1 + CTLA-4		Yes	peripheral neuropathy	2
31	79.9	Male	renal	Yes	nivolumab	PD-1		Yes	peripheral neuropathy	2
32	63.7	Female	renal	No	durvalumab + tremelimumab	PD-L1 + CTLA-4		Yes	myositis	4
33	75.0	Male	skin	Yes	nivolumab + ipilimumab	PD-1 + CTLA-4		Yes	myasthenia/myositis	4
34	55.7	Female	skin	Yes	nivolumab + pembrolizumab + relatlimab	PD-1 + LAG-3		Yes	peripheral neuropathy	4
35	55.3	Male	skin	Yes	pembrolizumab	PD-1		Yes	encephalitis or cerebellitis	4
36	55.4	Male	renal	No	nivolumab + ipilimumab	PD-1 + CTLA-4		Yes	peripheral neuropathy	3
37	61.9	Female	colorectal	Yes	dostarlimab	PD-1		Yes	myasthenia gravis	1
38	81.3	Female	skin	Yes	nivolumab + ipilimumab	PD-1 + CTLA-4		Yes	Sensory ganglionopathy	2
39	65.2	Male	skin	Yes	nivolumab + ipilimumab	PD-1 + CTLA-4		Yes	vestibulitis	2
40	54.8	Female	breast	Yes	atezolizumab	PD-L1		Yes	other	4
41	67.2	Male	skin	Yes	nivolumab + ipilimumab	PD-1 + CTLA-4		Yes	vestibulitis	3
42	63.8	Male	renal	No	pembrolizumab	PD-1		Yes	myasthenia gravis	2
43	64.6	Male	skin	Yes	nivolumab + ipilimumab	PD-1 + CTLA-4		Yes	peripheral neuropathy	3
44	48.5	Female	skin	Yes	nivolumab + ipilimumab	PD-1 + CTLA-4		Yes	aseptic meningitis	3
45	60.1	Female	skin + breast	Yes	nivolumab + ipilimumab	PD-1 + CTLA-4		Yes	peripheral neuropathy	3
46	74.3	Male	skin	Yes	nivolumab + ipilimumab	PD-1 + CTLA-4		Yes	Sensory ganglionopathy	3
47	58.7	Male	skin	Yes	nivolumab + ipilimumab	PD-1 + CTLA-4		Yes	mmm	4
48	76.0	Female	skin	Yes	pembrolizumab	PD-1		Yes	myelitis	3
49	52.1	Male	skin	Yes	nivolumab + ipilimumab	PD-1 + CTLA-4		Yes	peripheral neuropathy	2
50	75.7	Male	skin	Yes	nivolumab + ipilimumab	PD-1 + CTLA-4		Yes	Sensory ganglionopathy	3
51	89.1	Female	skin	Yes	pembrolizumab	PD-1		Yes	myasthenia gravis	2
52	70.6	Male	lung	Yes	pembrolizumab	PD-1		Yes	myasthenia/myositis	4
53	79.3	Male	hodgkin lymphoma	Yes	pembrolizumab	PD-1		Yes	peripheral neuropathy	3
54	55.4	Male	skin	Yes	nivolumab + ipilimumab	PD-1 + CTLA-4		Yes	encephalitis or cerebellitis	3
55	67.0	Male	lung	Yes	pembrolizumab	PD-1		Yes	peripheral neuropathy	3
56	73.6	Male	skin	Yes	pembrolizumab	PD-1		Yes	mmm	5
57	61.7	Female	skin	No	pembrolizumab	PD-1		Yes	peripheral neuropathy	3
58	78.2	Male	skin	Yes	ipilimumab	CTLA-4		Yes	peripheral neuropathy	4
59	68.3	Female	skin	Yes	nivolumab + ipilimumab	PD-1 + CTLA-4		Yes	peripheral neuropathy	3
60	76.5	Male	gastrooesophageal	No	nivolumab	PD-1		Yes	mmm	3
61	60.9	Male	sarcoma	Yes	nivolumab + ipilimumab	PD-1 + CTLA-4		Yes	other	4
62	58.2	Male	skin	Yes	pembrolizumab	PD-1		Yes	Sensory ganglionopathy	4
63	54.5	Male	colorectal	Yes	pembrolizumab	PD-1		Yes	myelitis	3
64	60.5	Female	skin	Yes	pembrolizumab	PD-1		Yes	mmm	3
65	86.0	Female	skin	Yes	pembrolizumab	PD-1	Dermatitis	No	peripheral neuropathy	1
66	89.0	Male	skin	Yes	pembrolizumab	PD-1	Arthralgia, Pneumonitis, Scleritis	No	peripheral neuropathy	3
67	70.0	Female	skin	Yes	pembrolizumab	PD-1	Hepatitis, Thyroiditis	No	peripheral neuropathy	2
68	57.0	Female	skin	Yes	nivolumab + ipilimumab	PD-1 + CTLA-4	Hepatitis, Thyroiditis	No	peripheral neuropathy	1
69	70.0	Male	skin	Yes	nivolumab + ipilimumab	PD-1 + CTLA-4	Colitis, Dermatitis, Pneumonitis	No	peripheral neuropathy	3
70	52.0	Female	skin	Yes	nivolumab + ipilimumab	PD-1 + CTLA-4	Hepatitis	Yes	encephalitis or cerebellitis	3
71	75.0	Female	skin	Yes	nivolumab + ipilimumab	PD-1 + CTLA-4	Dermatitis	Yes	peripheral neuropathy	3
72	67.0	Male	skin	Yes	nivolumab + ipilimumab	PD-1 + CTLA-4	Arthralgia	No	peripheral neuropathy	3
73	64.0	Male	skin	Yes	nivolumab + ipilimumab	PD-1 + CTLA-4	Hepatitis	Yes	peripheral neuropathy	3
74	55.0	Male	skin	Yes	nivolumab + ipilimumab	PD-1 + CTLA-4		Yes	peripheral neuropathy	3
75	63.0	Male	skin	Yes	nivolumab + ipilimumab	PD-1 + CTLA-4	Pneumonitis, Thyroiditis	No	peripheral neuropathy	1
76	62.0	Male	skin	No	pembrolizumab	PD-1		Yes	encephalitis or cerebellitis	4
77	77.0	Male	skin	No	pembrolizumab	PD-1		Yes	myasthenia gravis	5
78	83.0	Male	skin	Yes	pembrolizumab	PD-1		Yes	myasthenia gravis	5
79	72.0	Male	skin	Yes	nivolumab + ipilimumab	PD-1 + CTLA-4	Pancreatitis, Skin Rash	Yes	peripheral neuropathy	4
80	27.0	Female	skin	Yes	nivolumab + ipilimumab	PD-1 + CTLA-4		Yes	myasthenia gravis	4
81	81.0	Female	skin	No	pembrolizumab	PD-1	Colitis	No	vestibulitis	2
82	81.7	Female	lung	Yes	nivolumab	PD-1		No	peripheral neuropathy	1
83	60.7	Male	renal	Yes	avelumab	PD-L1		No	unclear	2
84	57.3	Female	breast	No	pembrolizumab	PD-1	Hepatitis	Yes	unclear	2
85	61.0	Male	skin	No	nivolumab + ipilimumab	PD-1 + CTLA-4		Yes	peripheral neuropathy	3
86	72.9	Female	lung	No	nivolumab + ipilimumab	PD-1 + CTLA-4		Yes	encephalitis or cerebellitis	2
87	83.3	Male	skin	Yes	pembrolizumab	PD-1		Yes	unclear	3
88	77.2	Male	lung	No	nivolumab + ipilimumab	PD-1 + CTLA-4		Yes	myasthenia/myositis	2
89	76.7	Male	skin	Yes	nivolumab + ipilimumab	PD-1 + CTLA-4		No	mmm	2
90	86.4	Male	skin	No	cemiplimab	PD-1	Hepatitits	Yes	myositis	5
91	36.8	Female	breast	No	pembrolizumab	PD-1		Yes	cranial neuropathy	3
92	62.8	Female	ovarian	No	nivolumab	PD-1		Yes	encephalitis or cerebellitis	2
93	81.0	Male	skin	Yes	pembrolizumab	PD-1	Myocarditis	Yes	mmm	3
94	77.8	Female	skin	Yes	nivolumab + ipilimumab	PD-1 + CTLA-4	Dermatitis	Yes	cranial neuropathy	2
95	84.5	Male	skin	Yes	pembrolizumab	PD-1		No	unclear	2
96	73.6	Female	skin	Yes	pembrolizumab	PD-1	Hepatitits	Yes	peripheral neuropathy	2
97	69.5	Male	skin	Yes	nivolumab + ipilimumab	PD-1 + CTLA-4	Dermatitis	Yes	myasthenia/myositis	3
98	68.4	Male	skin	No	pembrolizumab	PD-1	Colitis	Yes	peripheral neuropathy + vestibulitis	2
99	75.2	Female	lung	No	atezolizumab	PD-L1	Dermatitis	No	unclear	2
100	76.8	Male	renal	No	pembrolizumab	PD-1		Yes	mmm	3
101	93.0	Female	skin	Yes	cemiplimab	PD-1		No	peripheral neuropathy	2
102	70.4	Female	lung	Yes	pembrolizumab	PD-1	Arthralgia	No	unclear	2
103	65.3	Female	breast	No	pembrolizumab	PD-1		Yes	cranial neuropathy	2
104	56.7	Male	skin	Yes	nivolumab + ipilimumab	PD-1 + CTLA-4		Yes	mmm	2
105	88.7	Male	skin	Yes	pembrolizumab + ipilimumab	PD-1 + CTLA-4	Dermatitis	Yes	myositis	4
106	43.1	Female	skin	Yes	nivolumab + ipilimumab	PD-1 + CTLA-4	Dermatitis	Yes	peripheral neuropathy	2
107	56.6	Male	skin	Yes	nivolumab + ipilimumab	PD-1 + CTLA-4		Yes	peripheral neuropathy	3
108	79.3	Male	skin	Yes	pembrolizumab	PD-1		Yes	myasthenia gravis	4
109	62.4	Male	skin	Yes	nivolumab	PD-1	Dermatitis	No	peripheral neuropathy	2

Table S2. Referrals for possible Neurological Immune Related Adverse Events to a Tertiary Neurology Service.

	Immune Checkpoint Inhibitor Mediated		
	**Yes**	**No**	**Unknown**
Patient count<br>(N, %)	37 (36.2%)	62 (60.8%)	3 (2.9%)
Age<br>(Mean SD)	64 (12)	65 (10)	65 (NA)
Sex<br>(M : F)	12 : 7 (18 missing)	17 : 13 (32 missing)	1 : NA (2 missing)
Diagnosis	Neuropathy: 16<br>CNS inflammation: 9<br>Myopathic: 8<br>Myasthenic: 4<br>Vestibulitis: 3	Coincidental: 25<br>Chemotoxicity: 17<br>Neoplastic: 6<br>Other irAE: 6<br>Other: 4<br>Non-neuro: 2<br>Paraneoplastic: 2	Coincidental: 2<br>Paraneoplastic: 1
Cancer	Melanoma = 24<br>Renal = 6<br>Lung = 4<br>Breast = 1<br>CRC = 1<br>Haem = 1<br>SCC = 1	Melanoma = 19<br>Unknown = 14<br>Renal = 6<br>Lung = 4<br>Ovarian = 4<br>SCC = 4<br>GI = 3<br>Thymic = 3<br>Breast = 2<br>Haem = 2<br>CRC = 1<br>Pancreatic = 1<br>Schwannoma = 1<br>Thyroid = 1<br>Urothelial = 1	Melanoma = 2<br>Unknown = 1
ICI	PD-1: 34<br>CTLA-4: 22<br>PD-L1: 2<br>LAG-3: 1	PD-1: 24<br>CTLA-4: 10<br>PD-L1: 6	PD-1: 1<br>CTLA-4: 1

The majority of referrals for possible N-irAE were deemed not to be related to ICI therapy (n = 62). A large proportion were coincidental, related to other cancer therapies (e.g. chemotoxicity), or the underlying cancer. This strengthens the argument that Neurology opinion is important in diagnosis, and therefore management.
